# Supplementary material for: Determinants of natural adult sleep: An umbrella review
Source: PLoS One. 2022 Nov 7;17(11):e0277323. doi: 10.1371/journal.pone.0277323 (PMC9639822; doi:10.1371/journal.pone.0277323)
Supplement: S1 File — This file contains the search string used for the selection of articles. (PDF) [file pone.0277323.s002.pdf]

Search strategy: Umbrella review determinants of natural adult sleep

A. PubMED

1) Concept 1: SLEEP

Title/abstract

sleep* OR asleep OR time in bed OR bed time OR bedtime OR night rest OR night awak* OR night wak* OR drowsin* OR somnolent*

Mesh

“Sleep” OR “Sleep wake disorders” OR “Sleep phase chronotherapy”

2) Concept 2: DETERMINANT

Title/abstract

determinant* OR associat* OR correlat* OR relation* OR relate* OR factor* OR predict* OR influenc* OR effect* OR mechanism* OR parameter* OR impact

3) Filter: SYSTEMATIC REVIEW

((review[tiab] OR "Review"[Publication Type] OR "Meta-Analysis as Topic"[Mesh] OR meta-analysis[tiab] OR "Meta-Analysis "[Publication Type] OR “Evaluation Studies”[Publication Type] OR “Evaluation Studies as Topic”[Mesh]) NOT ("Letter"[Publication Type] OR "Editorial"[Publication Type] OR "Comment"[Publication Type]))

B. PsycInfo

S1: Thesaurus terms (equivalent MESH TERMS)

DE "NREM Sleep" OR DE "REM Sleep" OR DE "Snoring" OR DE "Napping" OR DE "Hypersomnia" OR DE "Insomnia" OR DE "Parasomnias" OR DE "Chronotype" OR DE "Fatigue" OR DE "Sleep" OR DE "Sleep Deprivation" OR DE "Sleep Onset" OR DE "Sleep Treatment" OR DE "Sleep Wake Cycle" OR DE "Sleep Wake Disorders" OR DE "Sleepiness"

S2 Subject term – sleep
SU sleep* OR asleep OR time in bed OR bed time OR bedtime OR night rest OR night awak* OR night wak* OR drowsin* OR somnolent*

S3: Title - sleep
TI sleep* OR asleep OR time in bed OR bed time OR bedtime OR night rest OR night awak* OR night wak* OR drowsin* OR somnolent

OR

S4: Abstract - sleep
AB sleep* OR asleep OR time in bed OR bed time OR bedtime OR night rest OR night awak* OR night wak* OR drow

AND

S5: Title – determinant
determinant* OR associat* OR correlat* OR relation* OR relate* OR factor* OR predict* OR influenc* OR effect* OR mechanism* OR parameter* OR impact

OR

S6: Abstract - determinant
determinant* OR associat* OR correlat* OR relation* OR relate* OR factor* OR predict* OR influenc* OR effect* OR mechanism* OR parameter* OR impact

AND

S7: review
AB review or meta-analysis or metareview or meta-review or meta review or literature review
OR
TI review or meta-analysis or metareview or meta-review or meta review or literature review

CINAHL

S1: MESH Terms
(MH "Sleep+") OR "sleep" OR (MH "Sleep Stages+") OR (MH "Sleep Disorders, Circadian Rhythm+") OR (MH "Sleep-Wake Transition Disorders+") OR (MH "Sleep Disorders, Intrinsic+") OR (MH "Sleep Deprivation") OR (MH "Insomnia") OR (MH "Sleep Disorders") OR (MH "Sleep, REM") OR (MH "Sleep Latency") OR (MH "Sleep Pattern Disturbance (Saba CCC)") OR (MH "Deep Sleep") OR (MH "Sleep Deprivation (Saba CCC)") OR (MH "Sleep Pattern Control (Saba CCC)") OR (MH "Sleep Hygiene") OR (MH "Sleep Pattern Disturbance (NANDA)") OR (MH "Sleep Enhancement (Iowa NIC)") OR (MH "Sleep and Rest Patterns (Omaha)") OR (MH "Sleep (Iowa NOC)") OR (MH "Polysomnography")

OR

S2: (TI (sleep* OR asleep OR time in bed OR bed time OR bedtime OR night rest OR night awak* OR night wak* OR drowsin* OR somnolent*)

OR
S3: AB (sleep* OR asleep OR time in bed OR bed time OR bedtime OR night rest OR night awak* OR night wak* OR drowsin* OR somnolent*)

S4 = S1 OR S2 OR S3

AND
S5: (TI (determinant* OR associat* OR correlat* OR relation* OR relate* OR factor* OR predict* OR influenc* OR effect* OR mechanism* OR parameter* OR impact)
OR
S6: AB(determinant* OR associat* OR correlat* OR relation* OR relate* OR factor* OR predict* OR influenc* OR effect* OR mechanism* OR parameter* OR impact)))

S7 = S5 OR S6

AND

S8: (TI ("review" or "meta-analysis" or “literature review” or “meta-review” or “meta review”) OR AB ("review" or "meta-analysis" or “literature review” or “meta-review” or “meta review”) OR MA (meta-analysis or systematic review) OR PT review)

S9 = S4 AND S7 AND S8

C. Web of Sciences / WoS

TS= combinatie van Title, Abstract, Author Keywords en Keywords Plus

QUERY:
(TS=(sleep* OR asleep OR time in bed OR bed time OR bedtime OR night rest OR night awak* OR night wak* OR drowsin* OR somnolent*))
AND
(TI=(determinant* OR associat* OR correlat* OR relation* OR relate* OR factor* OR predict* OR influenc* OR effect* OR mechanism* OR parameter* OR impact))

AND
(TS=(review or meta-analysis or literature review or meta-review or meta review))

OR

(TS=(sleep* OR asleep OR time in bed OR bed time OR bedtime OR night rest OR night awak* OR night wak* OR drowsin* OR somnolent*))
AND

(TI=(determinant* OR associat* OR correlat* OR relation* OR relate* OR factor* OR predict* OR influenc* OR effect* OR mechanism* OR parameter* OR impact))

AND DOCUMENT TYPES: (Review)

D. Embase

exp sleep/ or exp slow wave sleep/ or exp sleep induction/ or exp nonREM sleep/ or exp sleep quality/ or exp circadian rhythm sleep disorder/ or exp sleep latency/ or exp sleep time/ or exp sleep deprivation/ or exp sleep therapy/ or exp sleep pattern/ or exp night sleep/ or exp REM sleep/ or exp sleep hygiene/ or exp sleep parameters/ or exp REM sleep deprivation/ or exp sleep waking cycle/

OR
(sleep* or asleep or time in bed or bed time or bedtime or night rest or night awak* or night wak* or drowsin* or somnolent*).ab,ti,kw.

AND

(determinant* or associat* or correlat* or relation* or relate* or factor* or predict* or influenc* or effect* or mechanism* or parameter* or impact).ti.

AND
(review or meta-analysis or literature review or meta-review or meta review).ti,ab.

E. Cochrane

#1 MeSH descriptor: [Sleep] explode all trees
OR
#2 (sleep* OR asleep OR time in bed OR bed time OR bedtime OR night rest OR night awak* OR night wak* OR drowsin* OR somnolent*):ti,ab,kw

AND

#3 (determinant* OR associat* OR correlat* OR relation* OR relate* OR factor* OR predict* OR influenc* OR effect* OR mechanism* OR parameter* OR impact):ti,ab,kw

filter:REVIEW
