# Supplementary material for: Determinants of natural adult sleep: An umbrella review
Source: PLoS One. 2022 Nov 7;17(11):e0277323. doi: 10.1371/journal.pone.0277323 (PMC9639822; doi:10.1371/journal.pone.0277323)
Supplement: Attachment — Submitted filename: Response to Reviewers.docx [file pone.0277323.s006.docx]

Dear Dr. Erlando

We are delighted to receive the opportunity to further improve our manuscript.

Herewith we submit the revised version of our manuscript entitled ‘Determinants of natural adult sleep: an umbrella review’.

We believe that we enhanced the contribution of our manuscript to scientific research by addressing all comments made by the reviewers. Detailed responses to the reviewers’ comments are provided below as well as an overview of all changes in references of the manuscript.

Of course, we remain at your disposal should you have any further suggestions for improvements of our manuscript.

On behalf of all authors,

Sincerely,
Nicole Philippens

# RESPONSE TO REVIEWERS

# Reviewer #1

I read with pleasure the thorough revision made by the authors to their paper “Determinants of natural adult sleep: an umbrella review”. From my part the authors have addressed almost all the points, there are only some minor aspects still requiring clarification or to be remarked:

**Comment 1.1**: The authors state that they did not exclude reviews based on the registration method of sleep. I suggest clarifying the following sentence as it may be unclear to what extent the “referring to” was considered as an exclusion parameter (page 8 lines 169-172): “Since we wanted to identify determinants of natural sleep, we excluded reviews referring to sleep regulation (e.g. endocrine system), symptoms or consequences of sleep or sleep deprivation (for example sleepiness or diminished cognitive performance due to sleep deprivation) or registration methods of sleep parameters (e.g. actigraphy, EEG, enquiries).”

**Response 1.1:** Thank you and glad to hear that you mostly agree with the revisions on our manuscript. We agree with the reviewer that the terminology “referring to” is multi-interpretable and therefore we used the following wording instead: “Since we wanted to identify determinants of natural sleep, we excluded reviews that **focus on** sleep regulation (e.g. endocrine system), symptoms or consequences of sleep or sleep deprivation (for example sleepiness or diminished cognitive performance due to sleep deprivation) or registration methods of sleep parameters (e.g. actigraphy, EEG, enquiries). [Page 8, line 207]

**Comment 1.2** I agree with the definition differentiating determinants and interventions. However, on the practical side it really does not change a lot. For example, when considering meditative activity many of the reviews-metanalyses cited described randomized controlled trials for these activities where the participants were asked how these activities impacted many aspects of their lives, including sleep. As a randomized controlled trial is not a thing a person normally does and as the participants knew these activities could influence sleep they should be considered also as intervention.

**Response 1.2**: We agree with the reviewer that the difference between randomized controlled trials and interventions is a thin line. An RCT provides participants with a strict regime in the experimental group, which is indeed not the same as routine behavior. The difference however, that has led to our decision to include these reviews, is that within an RCT all other factors (except the determinant) are controlled within all reasonableness. An intervention, however, typically consists of multiple components that make it hard or even impossible to single out the effect of one determinant/factor. To address this issue properly we added the following text to the Discussion section (bold section): “An important area of concern and a limitation is that determinants are interrelated, which means that the relationship between determinants and sleep outcomes is not strictly linear, but most likely more complex. **This interrelatedness is one of the reasons we excluded studies on sleep interventions as it is difficult to single out the impact of one individual factor as a determinant.”** [Page 37, line 905]

In addition, we added a suggestion for future research on intervention studies on sleep as a different and valuable perspective. [Page 40, line 976].

**Comment 1.3**: I agree that some demographic confounders cannot be excluded as they were already included in the reviews-metanalyses, and I totally agree with the incorporated specification in the manuscript. However, it should be also pointed out that BMI is indeed target of sleep interventions, as weight control is the first line action to be suggested when addressing Obstructive Sleep Apneas.

**Response 1.3:** We agree with the reviewer that BMI has a profound effect on sleep and weight control should be an important focus of interventions by health practitioners supporting overweight clients with sleep improvements. We therefore added the following text lines to the discussion section: “Regarding weight (or BMI) it should be noted that overweight has in many cases a direct relation with obstructive sleep apneas, thereby severely impacting sleep quality. Health practitioners supporting overweight patients should address weight control as a first line of action. [Page 36, line 868]

**Comment 1.4:** Finally, the authors have both stated in revising their work that a) “this is a review which could be either used to support the practical use of the knowledge on sleep determinants in day-to-day practice of health professionals in a primary care setting” and b) “the review can be merely seen as a starting point for further research”. As a last suggestion, I would encourage the authors to focus on one of these goals, taking into consideration all the revisions, as again, with a single general work it could be highly difficult to tackle both points.

**Response 1.4**: Our manuscript would indeed benefit from one objective only. Our main objective is to support the practical use of the knowledge on sleep determinants in day-to-day practice of health professionals in a primary care setting. We later on mention that the review can be seen as a starting point for further research. The reason behind this remark is not to add another objective but to make readers aware of the fact that revisions of this umbrella review are to be expected.

To further clarify this focus on the practical use for health professionals we added the following text lines (we kindly refer to comment 4.2 as it is addressing this topic as well):“This umbrella review provides an overview of the current stand on research on sleep determinants, useful in day-to-day practice of health professionals addressing sleep issues with their patients. To enhance the practical use of the sleep determinants in daily practice, we recommend the presentation of the determinants in a well-structured and summarizing model, providing a subject for further research. Since sleep research is a trending topic it reflects the current availability of reviews on the determinants of sleep in natural circumstances but further research is to be expected.” [Page 39, line 956]

# Reviewer #2:

**Comment 2.1;** The authors addressed all the concerns raised by my revision except one. Namely, in my previous revision, I commented that:
"Considering personality, attachment style, sexual orientation and psychological dispositions in general as socio-economical determinants is questionable and the authors do not argument on it nor report on which bases they made that decision. It is my opinion that psychological characteristics should be distinguished from socio-economic characteristics. Moreover, the reference the authors cite for supporting their categorization (i.e. Health NSWDo. Public Health Classifications Project–Determinants of Health. Phase 2 Two: Final Report. 2010) does not include psychological characteristics/dispositions within the socio-economic determinants of health"

The authors' answer is:
"We agree with the reviewer that the term ‘socio-economic’ in general does not refer to personal psychological characteristics. However, the Public Health Classifications Project–Determinants of Health defines social determinants as determinants that influence health, including social, cultural, and gendered roles, religious belief or spirituality, health cognition; and other societal contributors such as social attitudes, class/ caste systems, community involvement and social and support systems. We belief that this definition leaves room for adding psychological aspects to this category. In addition, the other categories of determinants of health (behavioral, biological and environmental) are less appropriate options. In order to clarify our decision, we added the following explanation: ‘Though in general the term socio-economic does not include personal psychological characteristics, the elaboration of this category within the health classification of determinants (e.g. social, cultural, and gendered roles, religious belief or spirituality, health cognition; and other societal contributors such as social attitudes, class/caste systems, community involvement and social and support systems) leaves room for including psychological-related determinants and makes it the most appropriate category within this classification (page 25 line 621)."

I do not agree that the definition of ‘socio-economic’ determinants given by the Public Health Classifications Project–Determinants of Health leaves room to include psychological determinants within the socio-economic category. I think that "behavioral" category is much more appropriate.

**Response 2.1**: Thank you for pointing out the fact that the header of this section does not cover the content. We feel that psychological dispositions are the pillars/foundation on which behavior is manifested but that it is not part of actual behavior itself. Based on the reviewer’s feedback, however, we now changed the header of the section to “**Personal and** socio-economic determinants of sleep” [Page 25, line 625] and added a short explanation in the introduction of the paragraph (text in bold):

**“Personal and socio-economic determinants** consist of personal, social and economic influences on sleep: in other words, the conditions in which people are born, grow, live, work and age that reflect on their sleep. **Though the health classification of determinants originally labeled this category as “socio-economic determinants”**, the elaboration of this category (e.g. social, cultural, and gendered roles, religious belief or spirituality, health cognition; and other societal contributors such as social attitudes, class/caste systems, community involvement and social and support systems) leaves room for including psychological-related determinants and makes it the most appropriate category within this classification. **The personal and socio-economic determinants** therefore not only include the social-economic situation of an individual but also include individual and personality related social concepts such as psychological dispositions. Sleep is a fundamental biological activity but can also be perceived as a complex social process in which the characteristics of the individual interact with the social environment.”[Page 25, line 626]

*Further adaptations in the manuscript (in bold):*

Results were categorized in four main categories: biological, behavioral, environmental and **personal**/socio-economical determinants. [Page 1, line 24]

Therefore, we clustered and present the sleep determinants in four categories analogous to the classification of the Public Health Classifications Project for Determinants of Health: biological, behavioral, (physical) environmental and **personal/socio-economic** determinants of sleep [3]. [Page 11, line 286]

Second, some reviews combine several determinants within one of the four categories (biological, behavioral, (physical) environmental or **personal**/socio-economical) but none of the reviews covers determinants in more than one category. [Page 12, line 309]

Social and cultural aspects of the environment are included in the category of **personal**/socio-economic determinants. [Page 21, line 542]

Table 6 provides an overview of all identified **personal** **and** socio-economic determinants of sleep. [Page 31, line 787]

Title of table 6: Overview of **personal** **and** socio-economic determinants of sleep [Page 31, line 789]

Header of the first column in table 6: **Personal and** socio-economic determinants. [Page 31, line 789]

We found a wide variety of sleep determinants that were clustered around four main categories: biological, behavioral, environmental and **personal**/socio-economic determinants. [Page 34, line 798]

**Personal and** socio-economical determinants impact sleep directly by positive social relations and positive psychological dispositions. [Page 34, line 802]

# Reviewer #3:

**Comment 3.1**: Having all reviewers' comments been thoroughly addressed, the manuscript ends up being much improved relative to the previous version.

**Response 3.1**; Thank you for acknowledging the improvements and for your contribution to them.

# Reviewer #4:

Unfortunately, the authors did not respond to the points raised satisfactorily.

**Comment 4.1**: Specifically, I asked to discuss the findings more proactively and the authors overlooked this comment by stating that this would fall outside the aims of the review.

**Response 4.1**:

Thank you for pointing out the lack of a general recap and proactive discussion of the results. We agree that our manuscript could benefit from pointing out the most important findings from our perspective and therefore we now added this in our Discussion section: “We found a wide variety of sleep determinants that were clustered around four main categories: biological, behavioral, environmental and personal/socio-economic determinants. Biological determinants are practically impossible to change but they can explain differences between individuals and life phases and provide as such valuable information on natural sleep developments. Research on environmental determinants is limited to specific categories such as light and noise. Personal and socio-economical determinants impact sleep directly by positive social relations and positive psychological dispositions. Moreover, they indirectly impact sleep by increased stress levels due to social and personal circumstances. Behavioral determinants provide the most promising approach to improve sleep though it is complex as behavior in general has many parameters and the effect changes with the specifics of these parameters. [Page 34, line 797]

**Comment 4.2;** Secondly, I asked for a "Research Agenda/Future Perspectives" section to be added. Although the authors have included this section, the points discussed are too broad and fail in providing relevant recommendations for future research.

**Response 4.2:** We agree with the reviewer that the points that are addressed in the research agenda are broad. In the text lines suggested below we formulated the research suggestions more specifically and proactively. Moreover, we added an explanation on the difficulty of specific research suggestions and in addition we added a practical suggestion for further development.

*Recommendations for future research*

This umbrella review provides an overview of the current stand on research on sleep determinants, useful in day-to-day practice of health professionals addressing sleep issues with their patients. To enhance the practical use of the sleep determinants in daily practice, we recommend the presentation of the determinants in a well-structured and summarizing model, providing a subject for further research.

Since sleep research is a trending topic it reflects the current availability of reviews on the determinants of sleep in natural circumstances but further research is to be expected. Given the broadness of the topic of sleep, we mainly address directions for further research instead of specific recommendations.

Our suggestions for future research are [Page 39, line 955]:

- More systematic reviews or meta-analyses on bedding/mattresses and on cognitive activity would greatly enhance the quality and usability of the overview.
- More effort into clear definitions of relevant sleep parameters.
- Several consecutive days should be considered when looking at sleep in research and practice.
- More research elaborating on the relation between the different sleep determinants and the effect on accompanying sleep parameters.
- More effort should be put into designing high-quality intervention studies on sleep.

# Changes in references:

## Change #1

Laar Mvd, Hadden B. Slapen als een oermens : wat de evolutie ons leert over een goede slaap. Eerste druk. ed. [Eindhoven]: Merijn van de Laar; 2021. [reference 1]

**Action:** replaced by “Hirshkowitz M. Normal human sleep: an overview. Medical Clinics of North America. 2004;88(3):551-65”

## In addition, we removed the reference “Litwiller B, Snyder LA, Taylor WD, Steele LM. The relationship between sleep and work: A meta-analysis. The Journal of applied psychology. 2017;102(4):682-99.” [reference 2] on this textline but the reference itself is still used in the Result section of the umbrella review.

**Rationale**: In line with comment 2.1 we used a more descriptive definition of sleep as suggested by the reviewer and changed the references accordingly.

## Change #2

Malik SW, Kaplan J. Sleep deprivation. Primary care. 2005;32(2):475-90. [reference 11]

**Action**: replaced by

- Irwin MR. Why Sleep Is Important for Health: A Psychoneuroimmunology Perspective. Annual Review of Psychology. 2015;66(1):143-72. [reference 10]
- Krause AJ, Simon EB, Mander BA, Greer SM, Saletin JM, Goldstein-Piekarski AN, et al. The sleep-deprived human brain. Nature Reviews Neuroscience. 2017;18(7):404-18. [reference 11]

**Rationale**: in line with comment 2.2 we used more recent articles.

## Change #3

Watson NF, Badr MS, Belenky G, Bliwise DL, Buxton OM, Buysse D, et al. Recommended Amount of Sleep for a Healthy Adult: A Joint Consensus Statement of the American Academy of Sleep Medicine and Sleep Research Society. Sleep. 2015;38(6):843-4.

**Action**: reference added.

**Rationale**: In line with commen 2.3 we added a remark on the consensus on sleep duration and we added the accompanying reference.

## Change #4

Finan PH, Goodin BR, Smith MT. The association of sleep and pain: an update and a path forward. The journal of pain : official journal of the American Pain Society. 2013;14(12):1539-52. [reference 42]

**Action**: Removed.

**Rationale**: In line with comment 1.6 we removed the determinant pain from the umbrella review which includes the reference to this determinant.

## Change #5

Peplau LA, Perlman D. Loneliness : a sourcebook of current theory, research and therapy. New York, N.Y., [etc.]: Wiley; 1982. [reference 125]

**Action**: reference added.

**Rationale**: This reference was already cited but accidentally omitted in the first version of the reference index.

## Change #6

Alonzo R, Hussain J, Stranges S, Anderson KK. Interplay between social media use, sleep quality, and mental health in youth: A systematic review. Sleep medicine reviews. 2021;56:101414. [reference 133]

Ohayon MM, Carskadon MA, Guilleminault C, Vitiello MV. Meta-analysis of quantitative sleep parameters from childhood to old age in healthy individuals: developing normative sleep values across the human lifespan. Sleep. 2004;27(7):1255-73. [reference 134]

**Action**: references added

**Rationale**: In line with comment 3.2 we added text and both references to the discussion section.
